# Fabrication and Characterization of Flexible CuI-Based Photodetectors on Mica Substrates by a Low-Temperature Solution Process

**DOI:** 10.3390/ma17205011

**Published:** 2024-10-14

**Authors:** Chien-Yie Tsay, Yun-Chi Chen, Hsuan-Meng Tsai, Kai-Hsiang Liao

**Affiliations:** Department of Materials Science and Engineering, Feng Chia University, Taichung 40724, Taiwan; weufh369@gmail.com (Y.-C.C.); a23585050@gmail.com (H.-M.T.); ikylovejust@gmail.com (K.-H.L.)

**Keywords:** metal halide, p-type semiconductor, cuprous iodide, zinc substitution, solution process, mica substrate, flexible UV photodetector

## Abstract

Both CuI and CuI:Zn semiconductor thin films, along with MSM-structured UV photodetectors, were prepared on flexible mica substrates at low temperature (150 °C) by a wet chemical method. The two CuI-based films exhibited a polycrystalline phase with an optical bandgap energy close to 3.0 eV. Hall effect measurements indicated that the CuI thin film sample had p-type conductivity, while the CuI:Zn thin film sample exhibited n-type conductivity, with the latter showing a higher carrier mobility of 14.78 cm^2^/Vs compared to 7.67 cm^2^/Vs for the former. The I-V curves of both types of photodetectors showed asymmetric rectification characteristics with rectification ratios at ±3 V of 5.23 and 14.3 for the CuI and CuI:Zn devices, respectively. Flexible CuI:Zn devices exhibited significantly better sensitivity, responsivity, and specific detectivity than CuI devices both before and after static bending tests. It was found that, while the optoelectronic performance of flexible CuI-based photodetectors degraded under tensile stress during static bending tests, they still exhibited good reproducibility and repeatability in their photoresponses.

## 1. Introduction

The evolving wearable optoelectronics and electronics would be primarily based on flexible devices and circuits combining active and/or passive devices and lightweight flexible substrates for potential new applications, including flexible displays, wearable solar cells, electronic skins, biomedical sensors, and flexible sensors and detectors [1,2,3]. Intrinsic cuprous iodide (CuI) is a unique metal halide composed of environmentally friendly elements [4,5]. It has a wide direct bandgap of 3.1 eV and exhibits moderate transparency in the visible spectrum at a thickness of about 100 nm [6]. CuI is one of the most promising p-type compound semiconductors, featuring large metal *p* orbitals and small electronegativity anions which effectively realize a delocalized valence band maximum (VBM) and have a smaller effective mass, resulting in higher hole mobility compared to other p-type oxide semiconductors [7]. Furthermore, its iodide anion (I^−^) has sufficient orbital overlap, allowing for a wide adjustable conductivity through chemical composition modifications and/or process method selection [8].

S. Inudo et al. indicated that the electrical resistivity for CuI thin films is in the range of 10^−2^–10^3^ Ωhm·cm and pointed out that the electrical properties of such films depend on the preparation approaches and process conditions [9]. CuI-based semiconductor and conductive thin films are widely utilized as conductors in solar cells, active channel layers in thin-film transistors, sensing layers in ultraviolet (UV) photodetectors, thermoelectric devices, and p-n heterojunction diodes [4,10,11]. Recently, they have also been applied in flexible UV photodetector applications. For example, Y. Huang et al. deposited a p-type CuI semiconductor film on a flexible PI substrate using the copper film iodination method at room temperature. They selected Ag as contact electrodes to fabricate a metal–semiconductor–metal (MSM)-structured photodetector which exhibited good optoelectronic properties and bending stability [8]. Results of M. Krishnaiah et al. suggest that the amorphous phase Sn-doped CuI (CuI:Sn) films prepared by a solution spin-coating method and annealed at 140 °C for 2 h could serve as a promising semiconductor sensing layer for flexible transparent photodetector applications, such as Sn-CuI devices manufactured on polyethylene terephthalate (PET) substrates [12]. N. Yamada et al. demonstrated that amorphous indium gallium zinc oxide/cuprous iodide (*a*-IGZO/CuI) double-layer stacks on polyethylene naphthalate/indium tin oxide (PEN/ITO) and polyethylene terephthalate/indium tin oxide (PET/ITO), fabricated by sputtering, functioned as transparent p-n heterojunction diodes with rectification ratios of approximately 10⁶ and greater than 10⁵ at ±2 V, respectively [13]. Y. Liu et al. combined n-type amorphous zinc tin oxide (*a*-ZTO) and p-type CuI to form a heterojunction diode on PET substrates and developed self-powered flexible UV photodetectors that exhibited fast response and durability in the flexed state [14].

Mica exhibits excellent flexibility due to its natural layered aluminosilicate structure and ultra-flat surface [15]. It has a high tolerance for temperatures of up to 600 °C and superior dimensional stability and impermeability to oxygen and water compared to typical thin polymeric material substrates such as polyethylene terephthalate (PET) and polyimide (PI) [16]. Additionally, it is compatible with various thin-film deposition methods and microfabrication techniques, making it an ideal substrate for flexible optoelectronic devices [17,18]. N.P. Klochko et al. deposited polycrystalline CuI semiconductor films with thicknesses ranging from 1.0 to 1.2 µm on both rigid glass and flexible mica substrates using a wet chemical synthesis method. They studied the Seebeck coefficient and thermoelectric power factors of these films, emphasizing their potential for thin-film solar thermoelectric generation applications [19]. Furthermore, they deposited nanostructured CuI thick films (approximately 1.5 µm) on glass, mica, FTO glass, and glass/FTO/ZnO nanoarrays using the Successive Ionic Layer Adsorption and Reaction (SILAR) method. These films were examined for their structural, electrical, and optical properties for the purpose of developing heterostructure backward diodes [20].

Our earlier research demonstrated that solution-processed CuI semiconductor thin films could transition from p-type to n-type conductivity after the incorporation of Zn^2+^ ions into CuI nanocrystals, leading to improved electrical properties. The CuI photodetectors exhibited significantly enhanced photoresponse characteristics compared to the pure CuI photodetectors under ultraviolet A (UVA) illumination. In particular, the photodetectors with 8 at% Zn substitution showed the highest response current of 2.05 × 10^−^⁴ A, the best responsivity of 722 mA/W and a specific detectivity of 1.51 × 10⁸ J [10].

Combining the performance and versatility of compound semiconductor optoelectronic devices with bendable and stretchable substrates could produce flexible devices for various applications. In this study, we combined mica substrates with pure CuI and the 8 at% Zn-substituted CuI (CuI:Zn) semiconductor thin films to develop flexible compound semiconductor thin films and UV photodetectors. We investigated the microstructural features and the optical and electrical properties of mica/CuI and mica/CuI:Zn thin-film samples. Furthermore, we compared the physical characteristics of CuI-based thin films deposited on glass and mica substrates. The study also included the fabrication and characterization of flexible CuI-based UV photodetectors with a planar metal–semiconductor–metal (MSM) structure and studied how tensile stress, introduced through a bending test, influences their optoelectronic performance.

## 2. Materials and Methods of Experiments

Both cuprous iodide (CuI) and 8 at% zinc-substituted cuprous iodide (CuI:Zn) semiconductor thin films were prepared on muscovite mica substrates by a spin-coating technique and, later, by low-temperature heat treatment. Flexible muscovite mica substrates with dimensions of 30 × 30 × 0.12 mm^3^ were cleaned in ethanol and distilled water using a sonicator for 10 min and then dried on a ceramic hot plate at 120 for 10 min prior to the deposition of CuI-based thin films. The CuI precursor solution was prepared by dissolving the CuI powder (Strem Chemicals, purity 98.0%) in 2-methoxyethanol (2-ME, Acros Organics, Geel, Belgium, purity 99.0%) and monoethanolamine (MEA, Acros Organics, purity 99.0%) followed by stirring at 60 °C for 3 h to form a clear solution. The ZnI_2_ powder (Alfa Aesar, Haverhill, MA, USA, purity 97.6%) was used as the Zn ion source material and the Zn substitution level in CuI ([Zn]/[Cu] + [Zn]) was determined at 8 at%. The molar ratio of 2-ME solvent to MEA stabilizer was determined to be 1:1 and the concentrations of CuI and CuI:Zn precursor solution were kept at 0.5 M.

The spin-coated process was carried out at 3000 rpm for 30 s. Each spin-coated solution film was then preheated at 120 °C for 10 min to evaporate the low-temperature solvent. After repeating the spin-coating and preheating processes once, the preheated films were annealed in an air atmosphere at 150 °C for 1 h to form CuI phase crystals and enhance the densification of the solution-processed compound thin films. To fabricate the flexible metal–semiconductor–metal (MSM)-structured photodetectors, the authors deposited a pair of nickel (Ni) interdigital electrodes with a thickness of 50 nm on the mica/CuI-based thin film samples by a vacuum thermal evaporation technique with a fine stainless-steel shadow mask. The width, spacing and length of the interdigital electrodes designed in the shadow mask were 90 μm, 165 μm, and 3 mm, respectively.

X-ray diffraction (XRD) measurements using the grazing incidence diffraction method were performed to identify the phases and crystal structures of the as-prepared mica/CuI-based film samples. A field emission scanning electron microscope (FE-SEM, Hitachi S4800, Tokyo, Japan) was used to observe the surface micrograph and cross-sectional microstructures of the obtained thin films. A double beam ultraviolet-visible (UV-Vis) spectrophotometer (Hitachi U-2900, Tokyo, Japan) was used to measure the light transmission characteristic to determine optical properties, including average transmittance, light-absorption coefficients, and optical bandgap energy. The electrical resistivity and Hall effect properties were measured at room temperature with an Ecopia HMS-3000 Hall measurement system (Gyeonggi-do, Republic of Korea) using the van der Pauw method under a 0.55 T magnetic field. The concentration and mobility of the major carrier were calculated from the Hall voltage, and the thickness of the corresponding CuI-based film was determined from the cross-sectional FE-SEM image.

**Figure 1:**
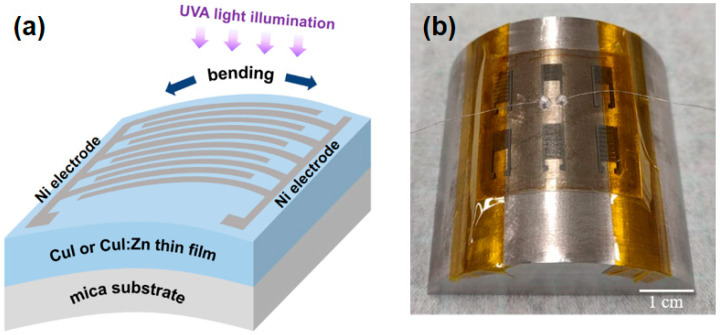
(**a**) Diagram illustrating the structure of a cuprous iodide (CuI)-based metal–semiconductor–metal (MSM) photodetector under UVA light illumination in the bent state; (**b**) photo of a CuI-based photodetector mounted on a convex stainless-steel base for static bending tests.

Current-voltage (I-V) and current-time (I-t) characteristics were measured using a Jiehan 5600 (Taichung, Taiwan) source measurement instrument in the dark and under UVA light illumination. Philips ultraviolet lamps (light source wavelength between 320 and 400 nm) provided an incident power density of 2.1 mW/cm^2^ to measure the transient photoresponse and photoswitching characteristics. In addition, we mounted the flexible photodetector on a convex stainless-steel base with a bending radius of 2.5 cm to measure the optoelectronic properties of the device under tensile stress (i.e., static bending test). Figure 1a shows a schematic diagram of the flexible MSM photodetector under static bending and UVA illumination, and Figure 1b shows the photo of the device as mounted on a convex stainless-steel base for static bending tests. To ensure the stability and reproducibility of the experimental values and results, at least three batches of each set of flexible CuI-based thin films or MSM photodetectors were developed in this study.

## 3. Results and Discussion

### 3.1. Characteristics of Spin-Coated CuI and CuI:Zn Thin Films

**Figure 2:**
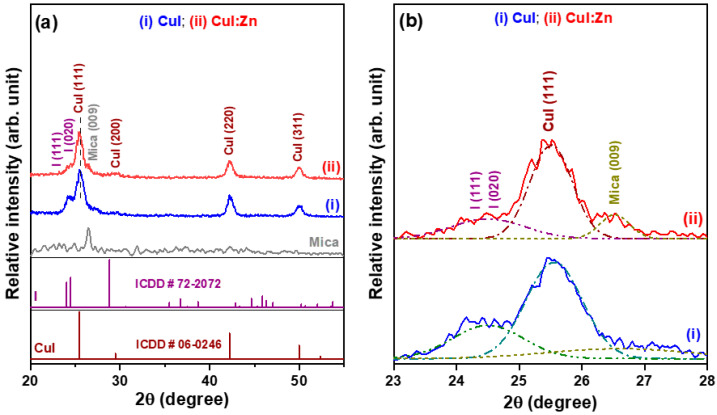
(**a**) X-ray diffraction (XRD) patterns of pure CuI and 8 at% Zn-substituted CuI (CuI:Zn) thin films deposited on mica substrates and (**b**) an enlarged graph of the CuI, I, and mica diffraction peaks in a narrow 2θ region for the corresponding compound thin film samples.

Figure 2a shows the XRD patterns of the pure CuI and 8 at% Zn-substituted CuI (CuI:Zn) thin films on mica substrates, revealing that both annealed CuI-based thin films exhibit a polycrystalline phase. Three X-ray diffraction peaks, along with a weak recorded signal, correspond to the (111), (220), (311), and (200) planes of the γ-phase CuI crystals with a cubic zinc blende structure, as identified by standard ICCD card No. 06-0246 [21]. The incorporation of Zn ions into CuI zinc blende lattices could induce compressive stress, causing a slight shift of the (111) plane XRD peak toward the lower 2θ diffraction angle region (Figure 2b). This occurs because Zn ions were substituted into Cu ion sites and/or filled partial Cu vacancies.

XRD examination also recorded a shoulder peak on the low 2θ diffraction angle side of the CuI (111) peak, which centered at around 2θ = 24.5°, which corresponds to the (111) and (020) planes of iodine (I) crystals, indicating the precipitation of the second phase (pattern (i) of Figure 2a). Additionally, a weak shoulder signal on the side of the high 2θ diffraction angle of the CuI (111) peak, centered at around 2θ = 26.4°, corresponds to the (009) plane of the mica substrate (pattern (i) of Figure 2a). Figure 2b shows that when Zn ions are incorporated into the CuI crystals, the XRD signals for the (111) and (020) planes of the iodine (I) phase (pattern (ii)) reduce to lower values compared to the undoped CuI crystals (pattern (i)). Additionally, a significant XRD signal associated with the (009) plane of mica is observed.

We found that the relative intensity of the XRD, calculated as (I_I(111)_ + I_I(020)_)/(I_CuI(111)+I(111)+I(020)_), decreased from 0.256 to 0.174 after incorporating 8 at% Zn ions into the CuI precursor solution. This result implies that the incorporation of the selected impurity dopant into the synthesis of polycrystalline CuI films can stabilize the growth of the CuI phase and suppress the precipitation of the I secondary phase. The spin-coated CuI-based thin films on glass also exhibit similar features (refer to the second row of Table 1). Scherrer’s equation is used to estimate the average crystallite sizes of the metal halide CuI and CuI:Zn thin films based on the XRD data, including the full widths at half-maximum and diffraction angles of the three major diffraction peaks ((111), (220), and (311) planes), as well as the X-ray wavelength of the diffractometer. The calculated results listed in Table 1 show that the CuI and CuI:Zn thin films have average crystallite sizes of 9.08 nm and 10.16 nm, respectively. The crystallite sizes of CuI-based thin films grown on mica substrates are larger than those of films deposited on glass because of differences in surface morphology and energy effects associated with the substrates. When Zn ions were introduced into solution-processed polycrystalline CuI thin films, the Zn^2+^ ions filled the Cu vacancies. This substitution of impurity ions resulted in a decrease in the concentration of Cu vacancies and an improvement in the crystallinity [10].

**Figure 3:**
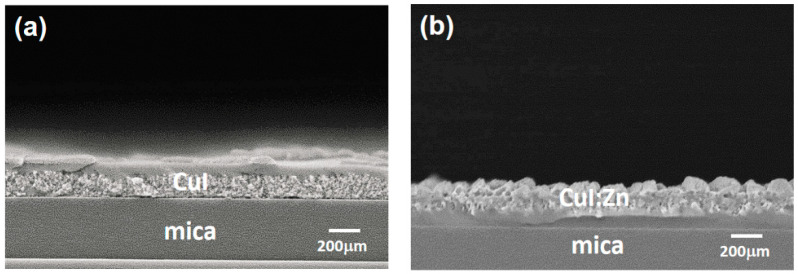
Cross-sectional micrographs of solution-processed (**a**) CuI and (**b**) CuI:Zn thin films on mica substrate obtained by field emission scanning electron microscopy (FE-SEM).

The thicknesses of the two CuI-based thin films were determined from cross-sectional FE-SEM micrographs, which are shown in Figure 3. The mean thicknesses of the CuI and CuI:Zn thin films grown on mica substrates were estimated to be 230 nm and 300 nm, respectively. It is well known that the thickness of spin-coated functional compounds and oxide films is strongly influenced by processing factors such as the speed of rotation, the viscosity of the coating solution, and the type of substrate. The CuI:Zn thin film is thicker than the pure CuI film, regardless of whether it is grown on glass or mica substrates.

**Figure 4:**
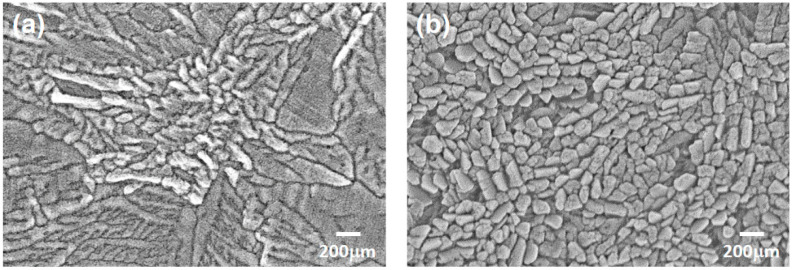
Micrographs of the surface morphology of (**a**) CuI and (**b**) CuI:Zn thin films deposited on mica substrate, as observed by FE-SEM.

The surface morphology of the two CuI-based thin film samples was observed using FE-SEM and shown in Figure 4. These micrographs reveal a granular microstructure with significant particle boundaries on the surfaces. The CuI:Zn thin film displays more uniform particle sizes compared to those of the pure CuI thin film. Previous reports have indicated that factors such as precursor concentration, deposition parameters, heat treatment and annealing temperatures, impurity doping elements and levels, as well as the processing environment can influence the nucleation and growth rates and the resulting microstructures and surface morphology of metal halide thin films prepared via spin coating. S. Sánchez et al. explained that the morphology of spin-coated perovskite thin films is controlled by nucleation and growth rates, as described by the classical LaMer growth curve [22].

**Figure 5:**
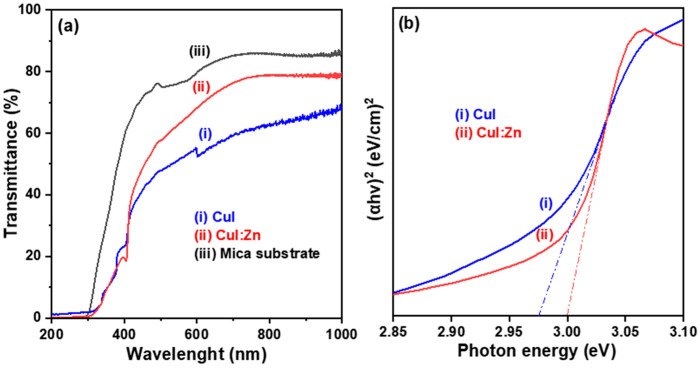
(**a**) Transmission spectra in the ultraviolet-visible (UV-Vis) region and (**b**) Tauc plots showing the variation in (*αhν*)^2^ versus photon energy (*hν*) for the CuI and CuI:Zn thin film samples.

The optical transmittance spectra of the mica substrate, mica/CuI thin film, and mica/CuI:Zn thin film samples are presented in Figure 5a to illustrate their transmission characteristics. Both CuI and CuI:Zn thin film samples exhibited low average transmittance, with values of 52.1% and 64.8%, respectively, in the visible region (400 to 800 nm). This low transmittance is due to the mica substrate, which has an average transmittance of only 78.8%, significantly less than the ~92% transmittance of commercially used display glass substrates. Furthermore, the transmittance spectra of the two CuI-based thin film samples showed sharp drops of around 411.5 nm, corresponding to excitonic band edge absorption, indicating a near-UV photoresponse.

The optical absorption coefficient (*α*(*λ*)) at a specific wavelength was calculated using the recorded transmittance data and film thickness (*t*) based on Beer’s Law. In wide bandgap compound and oxide semiconductors (*E_g_*~3 eV), optical energy transitions can occur as direct transitions between the valence band and conduction band of the energy bane. Consequently, we can evaluate the optical bandgap (*E_g_*) with direct allowed transitions for metal halide CuI and CuI:Zn semiconductor thin films using the following Tauc’s relation [21]:

(1) $$(\alpha h\nu )={A(h\upsilon -E_{g})}^{n}$$

where α represents the absorption coefficient, which is a function of wavelength, *α*(*λ*), the term *h* stands for Planck’s constant, *E_g_* denotes the optical bandgap of a semiconductor material, *ν* is the frequency of the incident photon, *A* is a proportionality constant that is related to the index of refraction as well as the effective masses of electrons and holes, and the Tauc exponent *n* is 1/2 in Formula (1) [23].

The Tauc plot shows the variation in the absorption energy (*αhν*)^2^ with the photon energy (*hv*), as calculated from the recorded transmittance data and estimated thickness of the thin film sample, shown in Figure 5b. The optical bandgap energy of the two CuI-based thin films was determined by extrapolating the tangential line near the absorption edge to the photon energy, *hv* (eV) on the *X*-axis. The determined optical bandgap energies for CuI and CuI:Zn thin films were 2.98 eV and 3.00 eV, respectively. These values are slightly lower than those of the corresponding CuI-based thin films deposited on glass substrates [10] and are close to the results reported by G. Lin et al. for undoped CuI thin films [24] and A. Liu et al. for Zn-doped CuI thin films [11].

Our previous study reported that incorporating Zn^2+^ ions into the CuI lattice could fill Cu vacancies and/or replace some Cu^+^ sites, providing free electrons. This increase in electron concentration led to a conversion of the conduction type in CuI-based materials from the p-type to the n-type [10]. The electrical properties, including the concentration of the major carrier (holes or electrons), the Hall mobility of the major carrier (*μ*), and the electrical resistivity (*ρ*) of the CuI and CuI:Zn thin films grown on mica substrates, are summarized in Table 1. The measured results of this study confirmed that the pure CuI thin film sample exhibited p-type conduction, while the CuI thin film sample showed n-type conduction. We found that the hole concentration in the CuI thin film grown on glass was 5.4 times higher than in the CuI thin film grown on mica; additionally, the electron concentration in the CuI:Zn thin film grown on glass was 16.3 times higher than in the CuI:Zn thin film grown on mica. Because the CuI-based thin films grown on mica exhibited a lower major carrier concentration compared to those grown on glass, they had higher electrical resistivity. Additionally, the compound thin films deposited on mica exhibited faster carrier mobility compared to those deposited on glass.

### 3.2. Photoresponse Performance of Flexible CuI-Based MSM Photodetectors

When the photodetector is attached to a concave steel base and subjected to compressive stress, the mica substrate could delaminate or crack. On the contrary, the attachment of the photodetector to a convex steel base subjects it to tensile stress. The smaller the radius of the convex base, the greater the stress on the component. In this study, the maximum tensile stress that the photodetector can withstand occurs with a convex base radius of 2.5 cm. During the bending test, the tensile strain (*ε_t_*) and tensile stress (*σ_t_*) generated in flexible devices can be estimated using the following two formulas [25]:

(2) $$\varepsilon_{t}=\frac{d}{2R}$$

(3) $$\sigma_{t}=\varepsilon_{t}\times \frac{E_{f}}{1-\nu^{2}}$$

where *d* is the thickness of the device, *R* is the bend radius and *E_f_* and *ν* are the Young’s modulus and the Poisson ratio of the device material. The Young’s modulus and the Poisson ratio of mica are 5.4 GPa [26] and 0.25 [27], respectively. Since the thickness of the mica substrate is much greater than that of the CuI-based thin films, the thickness effect of the CuI-based thin films can be ignored. The calculated tensile strain and tensile stress in the bent CuI-based MSM photodetectors are 2.4 × 10^−3^ and 1.4 × 10^−2^ GPa, respectively.

**Figure 6:**
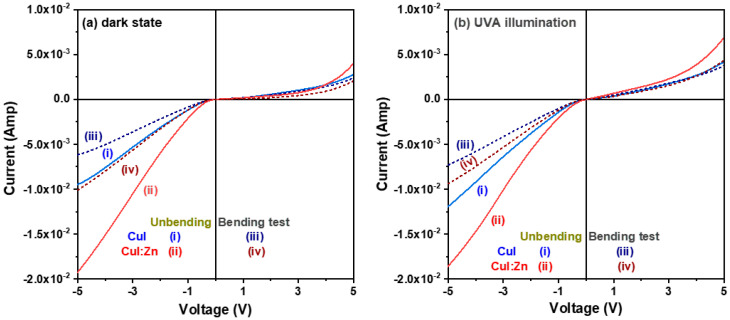
Comparison of the current-voltage (I-V) characteristics of CuI and CuI:Zn MSM photodetectors fabricated on mica substrates both with and without a bending test (**a**) in the dark and (**b**) under UVA light illumination.

The optoelectronic performance of a photodetector is directly related to the electrical characteristics and optical bandgap energy of its semiconductor sensing layer. Figure 6a,b compare the current–voltage (I-V) characteristic curves for CuI and CuI:Zn MSM photodetectors under an applied bias voltage across two Ni interdigitated electrodes, with a sweep from −5 V to +5 V, both in unbending and static bending states, in a dark environment and under UVA illumination. The I-V characteristic curves of the two CuI-based photodetectors exhibited an asymmetric rectification feature with rectification ratios at ±3 V of 5.23 for the CuI device and 14.3 for the CuI:Zn device (Figure 6a). The pure CuI device exhibited a lower dark current than the CuI:Zn device at both forward and reverse bias voltages in the dark environment (Figure 6a) due to the lower concentration of the major carrier in the p-type CuI semiconductor layer. When the CuI-based semiconductor sensing layers of the photodetectors were illuminated with UVA light, the semiconductor layers absorbed the light energy, leading to the creation of electron–hole pairs and photogenerated carriers, which formed a photocurrent when an external bias voltage was applied between the two Ni interdigitated electrodes. According to Figure 6a,b, the photoresponses (I_UVA_/I_dark_) for CuI and CuI:Zn devices at 0.5 V were 2.0 and 4.0, respectively.

Furthermore, the measured current for both CuI-based devices degraded after tensile stress was applied during static bending both in a dark environment and under UVA light illumination. We found that the rectification ratios for the CuI and CuI:Zn devices decreased by 25.62% and 5.60%, respectively, after the bending test was performed. Additionally, the turn-on voltage slightly reduced from 2.90 V to 2.85 V for the former and decreased from 14.3 V to 13.5 V for the latter. Despite the measured dark current and photocurrent being decreased, the photoresponse increased 16.26% and 64.68% for the CuI and CuI:Zn devices during bending test. Most functional compounds and oxide films used in optoelectronic devices are inorganic and inherently brittle, possibly causing microcracks to form inside the films when bent or stretched. These microcracks can disrupt the films’ microstructures, degrading their physical properties and severely affecting device performance [3].

**Figure 7:**
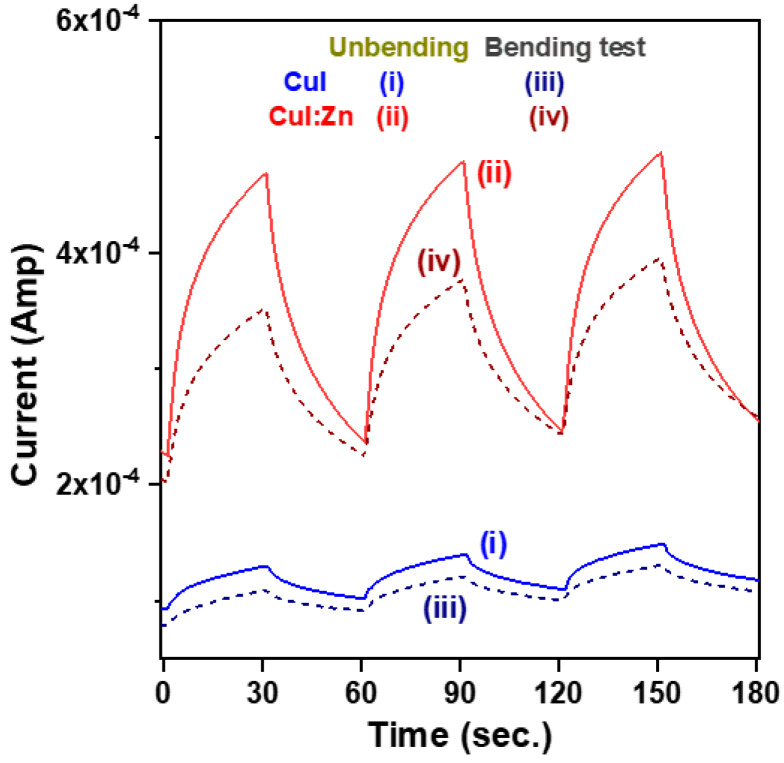
Photoresponse characteristics as a function of light exposure time for CuI and CuI:Zn MSM photodetectors fabricated on mica substrates and tested with and without bending under an applied bias voltage of 0.5 V. The UVA light was periodically turned on and off with a 30 s interval.

Figure 7 shows a comparison of the time-resolved photoresponse characteristics of CuI and CuI:Zn MSM photodetectors before and after static bending tests under UVA light illumination with a bias voltage of 0.5 V. To investigate the dynamic photoresponse characteristics of the two CuI-based MSM photodetectors, we conducted three consecutive on/off switching cycles both without and with bending tensile stress. The measured results revealed that both the CuI and CuI:Zn MSM photodetectors exhibited excellent photoswitching characteristics, demonstrating operational stability, repeatability, and reproducibility (I-t curves (i) and (ii) in Figure 7). When subjected to tensile stress in the photodetector, the recorded current decreased significantly, but the devices still maintained good photoswitching behavior (I-t curves (iii) and (iv) in Figure 7). Y. Duan et al. proposed that, when the devices are subjected to stress, mechanical displacement alters the charge distribution and generates a dipole moment, which reduces the depletion width and the electric field intensity in the space charge region. This leads to a decrease in the concentration of photogenerated carriers and reduces the separation efficiency of electron–hole pairs in the depleted layer, ultimately resulting in a reduction in the photocurrents and light responsivity of photodetectors [28].

The optoelectronic performance of CuI and CuI:Zn MSM photodetectors was evaluated using several photoresponse parameters, including photoconductivity gain (*G_ph_*), sensitivity (*S*), responsivity (*R*), specific detectivity (D*), and external quantum efficiency (*EQE*), and based on the measured results for current variation with irradiation time [29,30].

Photoconductivity gain (*G_ph_*) is defined as the ratio of light current (I_light_) to dark current (I_dark_) and is expressed as:

*G_ph_* = I_light_/I_dark_, (4)

The photocurrent (I_ph_) is the difference between light current (I_light_) and dark current (I_dark_). Sensitivity (*S*) is defined as the ratio of photocurrent (I_ph_ = I_light_ − I_dark_) to dark current (I_dark_) as given by the following formula:

*S* = I_ph_/I_dark_, (5)

Responsivity (*R*) is defined as the photocurrent (I_ph_) per unit of incident optical power (P_opt_) and is given as follows:

*R* = I_ph_/P_opt_, (6)

The unit of incident optical power (P_opt_ = P × A_0_) is obtained by multiplying the incident optical power density (P = 2.1 mW/cm^2^) by the effective irradiation area of the MSM photodetector (A = 19.308 mm^2^). Specific detectivity (D*) is the ability of the photodetector to detect the smallest signal expressed by

D* = R × (A_0_/2eI*_dark_*)^1/2^ (7)

where *e* is the electron charge. Additionally, the external quantum efficiency (*EQE*) measures the efficiency of converting photons into electrons. The ratio of photogenerated carriers collected by the photodetector electrodes is quantified by the number of incident photons on its surface.

*EQE* = (*hc*/*eλ*) × *R* (8)

where *h* is Planck’s constant, *c* is the speed of light, *e* is the electron charge, and *λ* is the incident light wavelength. Table 2 lists the calculated results for five photoresponse parameters to examine the impact of static bending on the quantified photoresponse performance. The CuI:Zn photodetectors exhibited better photoresponse properties than those of the pure CuI photodetector across all five evaluated performance parameters both before and after the static bending test. Furthermore, each photoresponse performance parameter degraded after static bending, and both types of CuI-based photodetectors were affected by continuous tensile stress and tensile strain. It is found that the magnitude of performance degradation for CuI:Zn photodetectors is more severe than that of CuI photodetectors. The possible reason is that the semiconductor layer thickness of CuI:Zn devices is about 23.33% thick compared with that of CuI devices, causing the former to bear greater tensile stress than the latter. The study contributed to the development of a flexible CuI-based photodetector through a low-temperature fabrication process and proposed great potential for flexible p-CuI/n-CuI:Zn homojunction optoelectronic devices.

## 4. Conclusions

Polycrystalline p-type CuI and n-type CuI:Zn semiconductor thin films were successfully grown on a flexible mica substrate by the spin-coating method and combined with a pair of Ni interdigitated electrodes to fabricate flexible MSM-structured UV photodetectors. A crack-free and dense surface morphology was observed for both the as-prepared CuI and CuI:Zn thin films. Their optical band gap was around 3.0 eV. The major carrier mobility of the n-type CuI:Zn semiconductor thin film is 14.78 cm^2^/V·s, which is nearly two times higher than that of the p-type CuI semiconductor thin film (7.67 cm^2^/V·s). Experimental results showed that the photoresponse characteristics, i.e., the light on/off switching behavior, of two kinds of CuI-based flexible UV photodetectors exhibited good repeatability and reproducibility and that the CuI:Zn photodetector had better photoelectric performance compared to the CuI photodetector. It was found that the photoresponse characteristics of the as-fabricated MSM photodetectors could be degraded after the static bending test because of the influence of tensile stress bearing on the semiconductor sensing layer.

## Figures and Tables

**Table 1:** Comparison of the structural characteristics, optical properties, and electrical properties of solution-grown CuI and CuI:Zn thin films deposited on glass and mica substrates.

Substrate	Compound Thin Film	Mean Film Thickness (nm)	^(a)^ Relative XRD Intensity of CuI and I Phases	^(b)^ Average Crystalline Size (nm)	Optical Bandgap (eV)	Carrier Concern (cm^−3^)	Hall Mobility (cm^2^/Vs)	Electrical Resistivity (Ω·cm)
Glass [10]	CuI	282	0.250	7.23	3.00	4.45 × 10^18^	2.86	4.86 × 10^−1^
	CuI:Zn	297	0.184	8.83	3.01	−4.34 × 10^18^	8.45	1.71 × 10^−1^
Mica	CuI	235	0.256	9.08	2.98	8.18 × 10^17^	7.67	9.96 × 10^−1^
	CuI:Zn	300	0.174	10.16	3.00	−2.66 × 10^17^	14.78	2.59 × 10^−1^

^(a)^ Calculated from the relative XRD intensity of the (111) plane of the CuI phase and the (111) and (020) planes of the I phase, as given by the formula (I_(111)_ + I_(020)_)/(I_CuI(111)_ + I_(111)_ + I_(020)_). ^(b)^ Estimated from XRD results using the Scherrer equation based on data from three major diffraction peaks.

**Table 2:** Comparison of photoelectric performance for CuI and CuI:Zn MSM photodetectors without and with static bending test.

Bending Testing	Sensing Layer Mater	Photoconductivity Gain (Unitless)	Sensitivity (Unitless)	Responsivity (mA/Watt)	Specific Detectivity (Jones)	External Quantum Efficiency (%)
Without	CuI	1.28	0.28	69.8	5.37 × 10^6^	2.34 × 10^4^
	CuI:Zn	1.95	0.95	575	2.85 × 10^7^	1.93 × 10^5^
With	CuI	1.20	0.2	44.1	3.58 × 10^6^	1.48 × 10^4^
	CuI:Zn	1.55	0.55	332	1.65 × 10^7^	1.11 × 10^5^

## Data Availability

The original contributions presented in the study are included in the article, further inquiries can be directed to the corresponding author.
